# Climate, immigration and speciation shape terrestrial and aquatic biodiversity in the European Alps

**DOI:** 10.1098/rspb.2022.1020

**Published:** 2022-08-10

**Authors:** Luiz Jardim de Queiroz, Carmela J. Doenz, Florian Altermatt, Roman Alther, Špela Borko, Jakob Brodersen, Martin M. Gossner, Catherine Graham, Blake Matthews, Ian R. McFadden, Loïc Pellissier, Thomas Schmitt, Oliver M. Selz, Soraya Villalba, Lukas Rüber, Niklaus E. Zimmermann, Ole Seehausen

**Affiliations:** ^1^ Eawag Swiss Federal Institute of Aquatic Science and Technology, 6047 Kastanienbaum/8600 Dübendorf, Switzerland; ^2^ Institute of Ecology and Evolution, University of Bern, 3012 Bern, Switzerland; ^3^ Department of Evolutionary Biology and Environmental Studies, University of Zurich, 8006 Zürich, Switzerland; ^4^ SubBio Lab, Department of Biology, Biotechnical Faculty, University of Ljubljana, 1000 Ljubljana, Slovenia; ^5^ Swiss Federal Institute for Forest, Snow and Landscape Research, 8903 Birmensdorf, Switzerland; ^6^ Department of Environmental Systems Science, Swiss Federal Institute of Technology in Zürich, 8092 Zürich, Switzerland; ^7^ Senckenberg German Entomological Institute, 15374 Müncheberg, Germany; ^8^ Institute of Biochemistry and Biology, University of Potsdam, 14476 Potsdam, Germany; ^9^ Naturhistorisches Museum Bern, 3005 Bern, Switzerland

**Keywords:** time for speciation, allopatric speciation, adaptive radiation, Pleistocene refugia, glacial species pump, European Alps

## Abstract

Quaternary climate fluctuations can affect speciation in regional biodiversity assembly in two non-mutually exclusive ways: a glacial species pump, where isolation in glacial refugia accelerates allopatric speciation, and adaptive radiation in underused adaptive zones during ice-free periods. We detected biogeographic and genetic signatures associated with both mechanisms in the assembly of the biota of the European Alps. Age distributions of endemic and widespread species within aquatic and terrestrial taxa (amphipods, fishes, amphibians, butterflies and flowering plants) revealed that endemic fish evolved only in lakes, are highly sympatric, and mainly of Holocene age, consistent with adaptive radiation. Endemic amphipods are ancient, suggesting preglacial radiation with limited range expansion and local Pleistocene survival, perhaps facilitated by a groundwater-dwelling lifestyle. Terrestrial endemics are mostly of Pleistocene age and are thus more consistent with the glacial species pump. The lack of evidence for Holocene adaptive radiation in the terrestrial biome is consistent with faster recolonization through range expansion of these taxa after glacial retreats. More stable and less seasonal ecological conditions in lakes during the Holocene may also have contributed to Holocene speciation in lakes. The high proportion of young, endemic species makes the Alpine biota vulnerable to climate change, but the mechanisms and consequences of species loss will likely differ between biomes because of their distinct evolutionary histories.

## 1. Background

Immigration, speciation and extinction are the three main processes underlying the assembly of biodiversity in island-like habitats [1–3]. The relative contribution of these processes depends on size, isolation and fragmentation of the region, ecosystem or habitat. For instance, immigration rates decrease with increasing isolation, extinction rates decrease with increasing area, and rates of *in situ* speciation increase with area, isolation and fragmentation [4–6]. The occurrence and interaction of these processes over geological history leave strong imprints in the contemporary structure of regional and local species assemblages, including phylogenetic structure and relatedness. The species age distribution (SAD) and the nature and degree of endemism are some of the resulting biodiversity features [7].

Endemism can arise through cladogenetic speciation (when an ancestral species diverges into two or more descendent species within a region), anagenetic speciation (a local or regional population or set of populations diverges from its progenitors outside the region) or through extinction everywhere else [3]. Recent cladogenetic and anagenetic speciation both result in neoendemic species, which are young species with geographically restricted distributions. Therefore, if cladogenetic and anagenetic speciations are important processes behind regional biodiversity assembly, a regional biota can be composed of many relatively young and closely related species. Non-endemic species, in turn, are generally more broadly distributed because they either have immigrated to the focal region from the outside or they have arisen in the focal region and had time to spread beyond it. Such non-endemic species are also expected to be older according to the ‘age and area’ hypothesis [8], which predicts that older species have had more time to disperse and become geographically more widespread. However, the ‘age and area’ hypothesis assumes biome stability (including climatic stability) and does not consider factors other than age that could in fact have strong effects on species range sizes, such as local extinction [9], physical or climatic barriers [10], habitat size, ecological versatility and evolutionary adaptability [11], dispersal ability [12] and ecological interactions [13]. The interaction of these factors can explain, for instance, why some species, despite being old, are geographically narrowly confined in the present time (geographical relicts or palaeoendemics) [14].

Mountain landscapes at lower and intermediate latitudes, such as those of the European Alps (hereafter the Alps), are hotspots of biodiversity and endemism [15,16]. In such environments, endemism and species radiations arise through the interaction of dispersal limitation with steep ecological gradients and often archipelago-like habitat structures [17–20]. In the Alps, multiple terrestrial taxa have undergone local radiations leading to the emergence of endemic clades in several groups such as flowering plants [21–23] and butterflies [24,25]. Some of the largest endemic radiations in European freshwaters also took place in or around the Alps, especially for amphipods [26–28] and fish [29,30]. These radiations occurred in habitats that are geographically isolated from similar habitats elsewhere, but surrounded by less isolated habitats, containing diverse assemblages of widely distributed taxa, such as mountain-tops surrounded by lowlands, or permanently cold, deep lakes isolated from other such lakes by the seasonally warm, shallow rivers.

**Figure 1.**
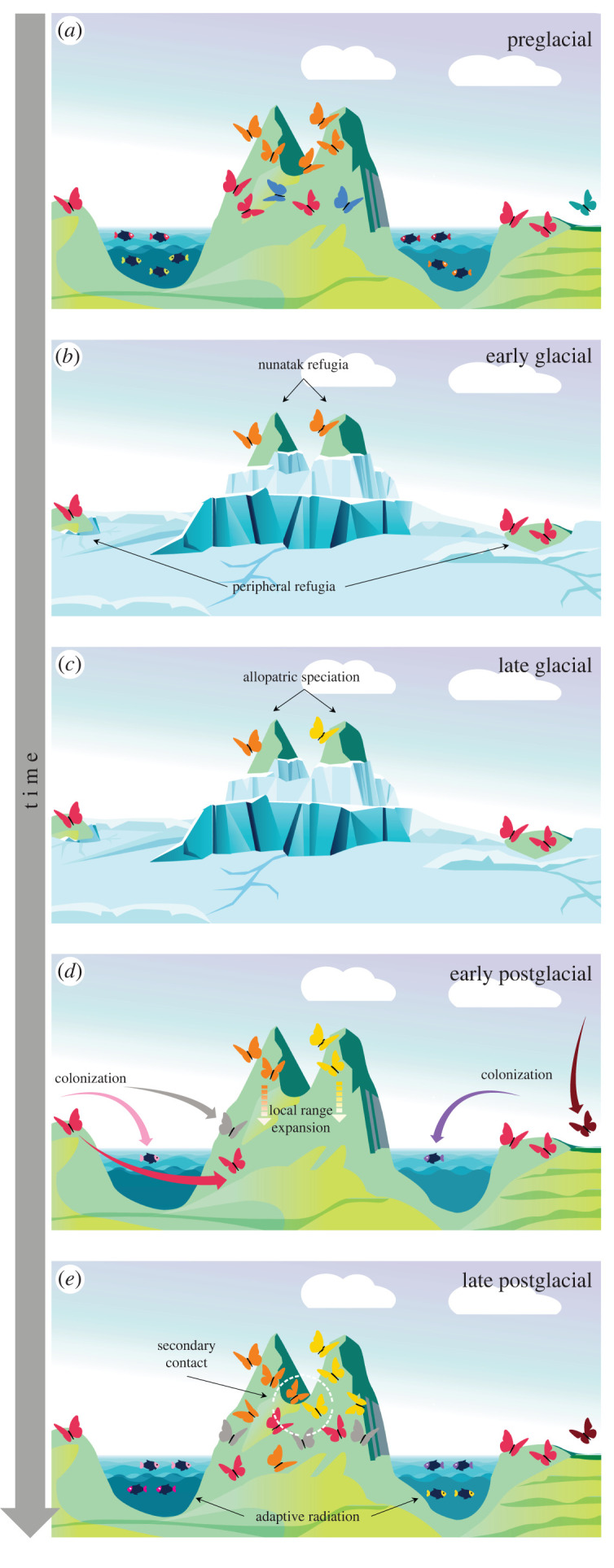
Evolutionary and ecological history of a hypothetical biodiversity assembly in an alpine-like system. (*a*) Biodiversity in a preglacial phase. (*b*) Early glacial phase: glacial periods erase freshwater habitats and fragment the terrestrial biome. Some populations survive in refugia and (*c*) can diverge into distinct species through allopatric speciation. (*d*) The retreat of glaciers opens up new, unoccupied habitats offering ecological opportunities for colonizers, (*e*) which undergo adaptive radiation, and niche space is filled up again. (Online version in colour.)

The Alps began to emerge at the end of the Cretaceous and during the Tertiary due to the collision between the African and European Plates [31], but geological uplift culminated in the Eo-Oligocene [32]. Early biodiversity in the Alps likely assembled through long-distance dispersal from older alpine regions, local range expansions or passive uplift during mountain-building [7,33,34]. Ancient evolutionary radiations are also expected to have taken place, but subsequent geological and climatic processes, such as glaciations, probably erased most of the footprints left by those ancient radiations [28]. The climatic and habitat instability driven by the Quaternary climate fluctuations was one of the most important historical factors shaping the modern biodiversity in the Alps [30,35–40], influencing immigration, speciation and extinction, and reshaping species abundance, range distribution, richness and genetic diversity patterns [41–43]. Neoendemic biodiversity in the Alps may have emerged through two alternative, non-exclusive mechanisms both driven by the succession of glacial–interglacial cycles: (i) fragmentation and isolation during glacial periods, the glacial species pump [44,45] and (ii) adaptive radiation during interglacial periods (hereafter adaptive radiation) [46] (figure 1). The former is a process in which allopatric speciation is accelerated via the isolation of small populations in glacial refugia, operating when the expansion of glaciers makes large areas of a species' range unavailable, but leaves isolated pockets of suitable habitat (figure 1*b*; [36,45,47,48]). It can therefore be expected that the glacial pump creates assemblages composed of species that originally emerged in allopatry but came into secondary contact more recently (figure 1*b–e*, butterfly example).

After each glacial maximum, the retreat of glaciers opens up new, unoccupied habitat in both terrestrial and aquatic ecosystems, which may facilitate early colonists’ *in situ* diversification through adaptive radiation [49] (figure 1*e*). Adaptive radiation takes place when an ancestor diversifies ecologically and phenotypically, giving rise to multiple species in short succession [50]. Adaptive radiation can also interact with the glacial pump mechanism as the postglacial expansion of populations may bring lineages together that had previously diverged in Pleistocene refugia [51]. Such secondary contact can facilitate the onset of adaptive radiation, either through ecological character displacement in sympatry occurring in response to competition [50], or through hybridization [52,53].

Although the SAD contains valuable information for investigating biodiversity assembly hypotheses and to understand biogeographic patterns [54], few studies have made use of it [40,55], and fewer even had investigated SADs from a multi-taxon perspective [7,56]. Here, we report on a comparative phylogenetic analysis to quantify SADs and the extent and type of endemism in aquatic and terrestrial ecosystems of the Alps. For terrestrial groups, we predicted that SADs support a scenario with resilience to cold glacial periods and potentially the glacial species pump for the origin of endemism, with species dating to the Pleistocene or before. Aquatic groups' SADs may indicate a prominent role of postglacial adaptive radiation. This is because during glacial maxima, high-altitude ranges in the terrestrial habitats became fragmented but not completely erased, whereas year-round open water bodies were entirely absent, with a few exceptions at the edge of the southern Alps [57]. Regarding non-endemic species, we do not expect differences in age structure among taxa because they tend to be widespread, are probably older and diversified on a wider geographical scale driven by processes that may have been decoupled from the climate dynamics of the Alps. Hence, we predict for all taxa that non-endemic species are older on average than endemics. Because dispersal ability, the distance between sink (new habitats that become available after glacial maxima) and sources of colonization, environmental stability and heterogeneity of habitat occupied vary between the taxa, the magnitude of the difference in SADs between non-endemics and endemics may be taxon dependent.

## 2. Methods

We focus on the European Alps, following previous delimitations of European high-mountain systems [58] but including the peripheral lowland areas, where the perialpine postglacial lakes are located, many of which emerged as deep fjords carved out by the glaciers. To select the taxonomic groups to be included in this work, we looked for lineages with reliable distribution data, robust dated phylogenetic trees that include most of the diversity of the given lineage, and/or sequence data for most of the recognized species, so that we could estimate and calibrate phylogenetic trees where none existed. We chose five major taxonomic groups to represent the terrestrial and aquatic alpine and pre-alpine biomes: amphipods, fishes, amphibians and butterflies (all nearly completely sampled), and 14 nearly completely sampled monophyletic clades of native flowering plants (Dicotyledons: Asteraceae: *Homogyne*, *Petasites*, *Tussilago*; Campanulaceae: *Campanula* clades 7–11 and 13–17 *sensu* [59], *Jasione*, *Physoplexis*; Caprifoliaceae: *Knautia*; Primulaceae: *Androsace* sect. *Aretia*, *Primula* sect. *Auricula*, *Soldanella*; Gentianaceae: *Gentiana*; Saxifragaceae: *Saxifraga* sect*. Saxifraga*; Monocotyledons: Cyperaceae *Carex* sect. *Phacocystis*; and Poaceae: *Festuca* sect. *Aulaxyper*).

To assemble SADs (stem age in million years, Myr), we combined published time-trees and our own estimates. We only used trees containing at least 50% of the valid species in the correspondent clade (electronic supplementary material, table S12). For the majority of butterflies and amphibians, we relied on two recently published super-trees [60,61], but for specific butterfly lineages underrepresented in the super-tree, we used complementary phylogenies [62–65]. To include Alpine species that were absent from these trees, we performed additional calibrations using secondary calibration points. For flowering plants, we used a set of published calibrated trees [66–74]*.* Amphipod trees were obtained from [28,75–78]. For fishes, we used a partial sequence (approx. 650 bp) of the *cytochrome c oxidase subunit I* (*COI*) gene (barcode region). Some of the sequences were generated in the present work, while others were taken from GenBank (electronic supplementary material, table S2). We included all species for which a barcode sequence was available. Calibrations were based on a molecular clock (1.2% genetic distance per Myr [79]). We are aware that this molecular clock derives from a relatively old study and on a biogeographic event (Panama Isthmus closure) that is not related to our target system. Yet, it still remains the most robust molecular clock available for the barcode region of fish. More details are in the electronic supplementary material.

### (a) Endemism and speciation mode

Species were considered endemic if they naturally occur only in the alpine and/or perialpine regions of the Alps. Speciation mode of endemic species was assigned to anagenetic speciation (an Alpine species diverged from its non-Alpine sister-species, but did not undergo *in situ* diversification) or cladogenetic speciation (Alpine species emerged through *in situ* diversification, with either the non-endemic sister being native to the region too, or the two or more sister-species all being Alpine endemics), following Rosindell & Phillimore [3] (electronic supplementary material, table S1). Therefore, if an endemic species was nested within a clade composed mainly of species that occur outside of the Alps, and its direct sister-species occurred only outside the Alps, we assumed anagenetic speciation. If the species was nested within a group mostly of species native to the Alps, we assumed cladogenetic speciation.

### (b) Comparisons of species age distributions

We performed permutation tests on the distribution of age estimates to identify differences in the SADs among and within major clades. We asked (i) whether age distributions differed between endemic and non-endemic species, overall and within each taxonomic group independently; (ii) whether SADs differed among taxonomic groups; (iii) whether SADs of non-endemics differed among groups and (iv) whether SADs of endemics differed among groups. When a difference was significant, we performed *post hoc* pairwise permutations to identify which distribution and taxonomic group (or groups) were distinct from one another after Bonferroni correction. These analyses were performed using the function ‘oneway_test’ of the library ‘coin’ [80] in R v. 4.0.2 1106 [81] with 10 000 resamples with a distribution approximated via Monte Carlo resampling.

### (c) Sensitivity to alternative divergence times

Although our work provides a powerful analysis of biodiversity assembly in the Alps, we are aware of some shortcomings. Our age estimates, by necessity, were assembled from different sources, and therefore no standardized methodology to infer and calibrate the phylogenies over all taxa was possible. For instance, the phylogenies used here can include a different set of markers and calibration strategies (e.g. fossils, secondary calibration or molecular clocks), which may introduce bias in our analyses. To investigate the potential impact that uncertainty in our estimates of species ages may have on our conclusions, we evaluate the 95% highest posterior densities (95% HPD). We first visually compared the minimum and maximum 95% HPD ages estimated for each species with the median values. Furthermore, for each species, we randomly picked an age from the 95% HPD, assuming the range to have a uniform distribution, and replotted the SAD for the group to assess if the patterns obtained with the median age are robust to random resampling (1000 times) from the 95% HPD of each species' ages. Given that fish phylogenies were built from a single short-sequence fragment (the barcode region), we also performed additional calibrations using a constant population tree prior for three lineages (*Coregonus*, *Salvelinus* and *Cottus*) that are known for having recently radiated (electronic supplementary material, Methods).

## 3. Results

**Figure 2.**
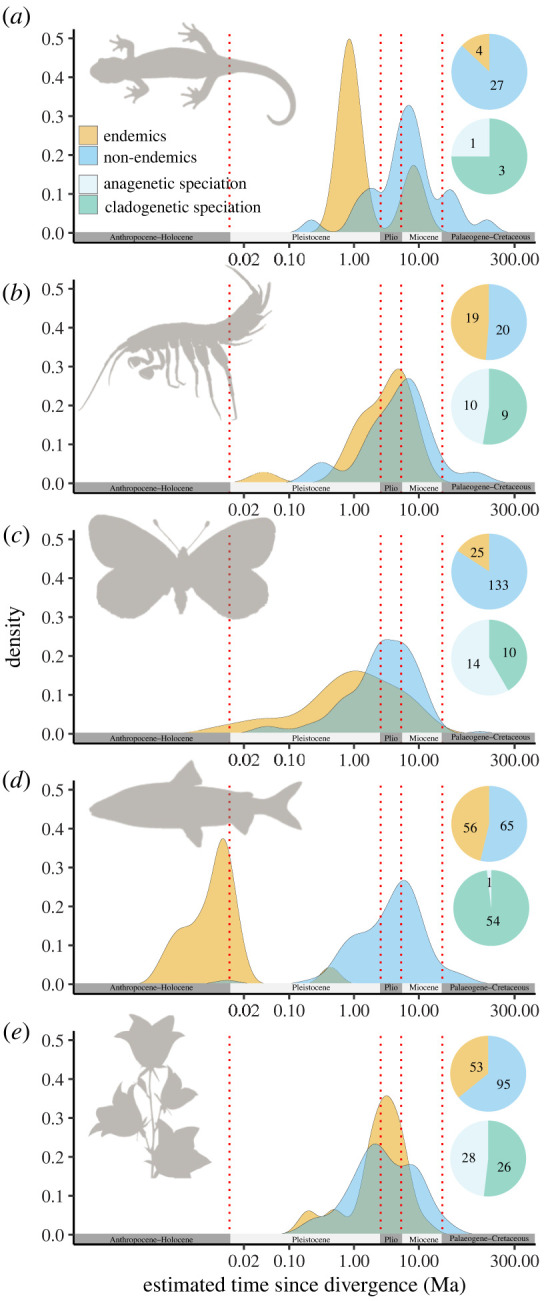
SAD of endemic and non-endemic species of (*a*) amphibians, (*b*) amphipods, (*c*) butterflies, (*d*) fish and (*e*) flowering plants. Pie charts show the proportion of endemic and non-endemic species as well as the proportion of endemic species that have emerged through cladogenetic or anagenetic speciation. (Online version in colour.)

A total of 497 species were included in our analyses: 39 amphipod, 121 fish, 31 amphibian, 158 butterfly and 148 plant species (electronic supplementary material, table S1). Approximately half of the fish and amphipod species were endemic to the European Alps (45% and 49%, respectively), whereas smaller fractions of 13%, 16% and 36% of the amphibians, butterflies and plants, respectively, were found to be endemic (figure 2). Around half of the endemic amphipods, plants and butterflies (53%, 52% and 41%, respectively) and the majority of the endemic amphibians and fish (75% and 98%, respectively) had arisen by cladogenesis (figure 2).

Based on the median species age, we found that around 95% of the extant, native species that now occupy the Alps, irrespective of endemism status, had emerged within the past 14 Myr (figure 2). Endemics were overall younger than non-endemics (*p*-value < 0.0001), and this was also true within each taxonomic group (electronic supplementary material, table S9). We found SAD of non-endemic species to be similar between taxonomic groups (figure 2), with most of the species ages spanning the Late Pleistocene to Early Miocene (90% fell between 0.3 and 17 Ma). The only significant difference between taxa was that non-endemic amphibians were older than non-endemic plants (*p* < 0.0005; electronic supplementary material, S10). SADs of endemic species were also similar among taxa (90% fell between 0.15 and 8 Ma), except for fish, which are younger than any other group of endemics (90% fell between 1.5 and 114 kyr; *p* < 0.0001; figure 2; electronic supplementary material, S11). Calibration of the lineages that have diversified after the Last Glacial Maximum (LGM) using a constant population tree prior also supported the postglacial radiation hypothesis (Trees in Data accessibility).

The plots based on random ages picked from the 95% HPD (electronic supplementary material, figure S1) corroborated the patterns. Even only considering the minimum ages, the data would still suggest only a small fraction of postglacial speciation in butterflies and plants. However, if we assumed the maximum ages, the data would imply that most of the endemic fish speciated before the LGM (electronic supplementary material, figure S2).

## 4. Discussion

We found that most of the non-endemic species of fish, amphipods, amphibians, butterflies and plants of the Alps emerged in the Middle Miocene. This is the time when central and southern Europe had first coalesced into a little continent that was beginning to have faunal exchange with Asia and Northern Europe. It was also the period of maximum geological uplift in the region that culminated with the formation of the Alps [32]. Fossil records from the Miocene indicate that close relatives of many of the modern taxa were already in the region, but many other taxa existed too that later went extinct in Europe but persist until today in Africa, especially among the fish [82,83]. Yet, it is the endemic species that showed the most intriguing patterns. Speciation timing involving endemics was dramatically different among taxa. While most of the Alp's endemics in the terrestrial groups originated in the Pleistocene, most endemic fishes arose after the LGM and re-establishment of permanent open water bodies in the formerly glaciated areas. Although there is some uncertainty around the youngest species age estimates from our calibrated trees for fish, a demographic study in one of the recently diversified lineages (*Cottus*) showed that the ancestor that gave rise to the modern radiation colonized the region only around 6.6 kyr ago (3.4–10.6 kyr) [39], supporting our results. Given that the vast majority of endemic fish are products of cladogenetic speciation, we suggest that the assembly process of the fish fauna of the Alps is dominated by an interaction between colonization from outside the region and postglacial adaptive radiation in lakes. By contrast, for the terrestrial groups, persistence through glacial climate cycles, the glacial species pump, and recent colonization from outside the region, seem to be the dominant mechanisms, as anagenetic speciation was more important in these taxa, and endemic richness assembled throughout the Pleistocene. Interestingly, we observed some postglacial speciation in butterflies, coinciding with the major mode in fish, but being dwarfed by the much larger Pleistocene mode in butterflies. However, an alternative scenario would be that sister-groups of extant terrestrial species became extinct at some point in time, meaning that extant species should be younger than we observe here, or that the absence of some extant species would also contribute to overestimating species age.

We suggest that common mechanisms underlie these contrasting patterns: (i) Quaternary climate fluctuations initiating allopatric species differentiation during cold stages and creating ecological opportunity for adaptive radiation during interglacial periods, (ii) variation among groups in their dispersal ability and associated rate of range expansion, and (iii) variation among biomes in the predictability and intensity of seasonal access to resources constraining ecological specialization and adaptive radiation.

### (a) Quaternary climate fluctuations

That most endemic fish are of postglacial origin, while endemics in other groups arose in the Pleistocene or earlier, is probably explained by different effects that the Quaternary climate oscillations had on freshwater versus terrestrial habitat availability [57]. Permanent open surface water habitats, as required by fishes, were absent in the glaciated parts of the Alps during the LGM, because lakes and river valleys were filled with glaciers. Therefore, the lack of endemic fish species older than 20 kyr on the northern and western flanks of the Alps is probably due to extirpation of populations across the region during the LGM. With the retreat of glaciers in the course of the Holocene, fish would then have returned to the region from refugia located in downstream sections of large rivers. For most species of temperate climates, these refugia were often far from the Alpine region, especially on the North face of the Alps, i.e. the lower Danube and lower Rhone [84]. This Holocene recolonization explains the large fraction of old, widespread and non-endemic fish species in the northern and western Alpine region.

The first fish to colonize after the LGM were probably species adapted to cold waters, such as salmonids and sculpins, that would have lived nearby in the rivers of the Pleistocene tundra downstream of the Alpine glacier shield, as they exhibit a remarkable ability to establish in postglacial freshwater habitats [85,86]. These fish would have encountered ecological opportunities in the emerging large, deep lakes in response to which they radiated into many distinct species due to strong divergent natural selection [87]. The selective forces driving such radiations are related to vacant niches associated with distinct lacustrine habitats in the deep fjord lakes [29,88,89]. This was likely the main process by which the young endemic species in three lineages, whitefish (*Coregonus* [88]), chars (*Salvelinus* [90]) and sculpins (*Cottus* [30]), diversified in perialpine lakes. The few old, relic endemic fish species in the region that date to prior to the Holocene are the lake herring *Alosa agone* and the Lake Garda trout *Salmo carpio.* These species are endemic to lakes in the south of the Alps, a region where probably not all lakes were completely glaciated during the LGM [57]. Therefore, these species may have originated during earlier interglacials, when southern perialpine lakes would have become extensive, and then found refugia during the LGM to persist to the present day [91]. That there are no young postglacial species among the non-endemic fish is perhaps due to insufficient time and connectivity between lakes to allow new species that arose in deep lakes elsewhere in Europe (e.g. in northern Germany and Scandinavia) in the Holocene to expand their range into the Alps or vice-versa.

Unlike fish, endemic amphipods, the second fully aquatic taxon in our data, emerged during or above all before the Pleistocene. Amphipod radiations in Europe are known for being rather ancient, predating the Pleistocene [28,92,93], while the youngest radiations probably are from the Pleistocene [94]. This corresponds to the paucity of postglacial speciation noticed here for this group across the Alps. The presence of many relatively old endemic amphipods in this region could be because, surviving in smaller water bodies than those required by fish, they may have persisted under ice cover, in glacier forefields [95], or in subterranean refugia, such as caves or groundwaters [26,28,94], habitats that were not completely erased by the Pleistocene glaciations.

Endemic species in the terrestrial groups are also older than endemic fish species, dating mostly to the Pleistocene or before, even if we consider the minimum age from the 95% HPD. Nunataks (mountain peaks that have never been glaciated [96]) and peripheral refugia surrounding the Alps are some of the types of Pleistocene refugia where many terrestrial taxa may have survived glaciations [40,48,97–105], such that extinctions during glacial cycles did not entirely wipe out the terrestrial biodiversity in the region. Although amphibians are in general semi-aquatic organisms, most species only require shallow open water bodies during spring and summer, and are terrestrial for the remainder of the year, while some lack an aquatic life stage altogether [106]. Therefore, amphibians likely found refugia within the Alpine region during glacial maxima [103,104]. In this way, fragmentation of the terrestrial habitat and consequently of populations during the Pleistocene might have accelerated allopatric speciation (Pleistocene species pump) in terrestrial organisms, explaining part of the endemic diversity in this realm. A similar phenomenon likely drove Pleistocene speciation in amphipods, as recently proposed [94]. Therefore, differential impacts of Quaternary climate fluctuations and the resulting glaciations on different biomes and taxa go a long way in helping to explain extant patterns of diversity and endemism in the region.

### (b) Dispersal ability

Dispersal ability often correlates negatively with rates of niche evolution and speciation [107–109]. Therefore, the large number of cases of postglacial speciation in fully aquatic taxa and its rareness in terrestrial taxa could be related to the dispersal rates imposed by the environments. Terrestrial environments, in general, offer less resistance to dispersal and range expansion than dendritic freshwater environments, both because of the higher dimensionality of the terrestrial landscape and as many terrestrial species have acquired adaptations for aerial dispersal, such as active flight in butterflies [110,111] or passive airborne propagation in plants [112]. Conversely, freshwater-bound taxa need to navigate the dendritic landscapes to disperse, making it more difficult to reach isolated habitats [113–115]. Given that, postglacial range expansion may have happened at a slower pace in entirely aquatic than in most terrestrial taxa. Faster recolonization by terrestrial taxa after glacial retreat was likely additionally facilitated by the proximity of refugia to the Alps (including inner-alpine refugia), as can also today be observed in contemporary glacier retreats [116]. Recent studies have shown indeed that many plant species rapidly and substantially expanded their range during postglacial periods [117–119]. Faster filling of emerging terrestrial habitats through colonization and range expansion left fewer opportunities and/or less time for the first colonizing species to undergo ecological speciation and adaptive radiation in response to ecological opportunity and selective forces. To test the relative importance of dispersal limitation versus other aquatic/terrestrial differences, future work could investigate aquatic taxa with strong aerial dispersal abilities, such as Odonata and other insects that spent most of their life cycle in freshwater and have short but highly dispersive terrestrial adult phases.

### (c) Seasonal and inter-annual environmental variation

Habitat stability and predictability at ecological time scales may have been additional factors explaining the larger number of Holocene speciation events only in lacustrine fish. Theory and models suggest that environmental fluctuations and stochasticity can reduce or inhibit ecological speciation because rapid variation in environmental conditions, both seasonal and inter-annual, makes specialization difficult and ecological speciation nearly impossible, especially when predictability is low [120,121].

Environmental conditions in terrestrial ecosystems at higher latitudes are highly seasonal and the onset and length of seasons vary dramatically from year to year, especially in high-mountain environments [122,123]. Aquatic ecosystems, on the other hand, are less seasonal [124,125]. This is especially true for the deeper zones of large and deep lakes, habitats that lack seasonality and year-to-year variation almost entirely and are nearly constant through the year [126,127]. Despite its low productivity, the longer growing and reproductive season and the more stable environment in deep lakes may create favourable conditions for individual specialization and ecological speciation. Moreover, in spite of greater temporal stability, deep lakes have steeper environmental gradients because pressure, light and temperature change faster with depth in water than with elevation in the terrestrial realm. This combination of unique properties of water may facilitate divergent selection leading to speciation and helps to explain the high frequency of ecological speciation in deep lakes, with sister-species being spatially very close to each other but occupying different water depths [89].

## 5. Final considerations

We suggest that the formation of the unique biota of the European Alps was driven by interacting mechanisms: non-random variation in Pleistocene population persistence, postglacial immigration, vicariant speciation during glacial maxima and adaptive radiation in the postglacial. These interacting mechanisms left distinct imprints on the species age structure of regional assemblages in the different biomes and their associated taxa. Historical factors (Quaternary climate fluctuations and Pleistocene refuge availability) impacted freshwater and terrestrial biomes in different ways, and contemporary ecological factors such as environmental stochasticity and dispersal limitations also vary between these biomes, shaping them very differently through ecological and evolutionary processes: high survival, fast recolonization but slow diversification in terrestrial, versus low survival, slow recolonization but fast diversification in aquatic systems. In the future, a broader taxonomic sampling, including other insect groups, mammals, birds and reptiles, as well as sampling alpine ecosystems in other parts of the world, may shed additional light on the generality of our findings.

Knowing the history of biodiversity formation is crucial for establishing strategies for conservation [128]. Our results confirm that the Alps is an endemism hotspot and improve our understanding of how this endemism emerged. Endemic species are range-restricted, show limited population size and are hence more vulnerable to climate change and other environmental changes than non-endemic species [129]. Because of that, they are of high concern for conservation. Even a comparatively small and transient disturbance of an ecosystem can lead to the extinction of young endemic species that evolved in adaptation to specific ecological conditions [130]. The sharp increase in extinction rates driven by human activity threatens the biodiversity of the Alps, and especially that of endemic species [131]. Therefore, this region, and alpine regions around the world, deserves greater attention to conserve both the regional biodiversity, as well as the eco-evolutionary processes that gave rise to it and that are required to continue operating if biodiversity is to be safeguarded in the longer term.

## Data Availability

Newly generated COI-sequences: GenBank accessions ON934039–ON934153. Phylogenetic data and DNA sequence alignments: available at Mendeley Data, https://dx.doi.org/10.17632/95n8kx8h54.1 [132]. The data are provided in the electronic supplementary material [133].
